# Residual and ecological risk assessment of heavy metals in fly ash from co-combustion of excess sludge and coal

**DOI:** 10.1038/s41598-021-81812-5

**Published:** 2021-01-28

**Authors:** Yao Tang, Jingshi Pan, Biqing Li, Suying Zhao, Liguo Zhang

**Affiliations:** 1 School of Mathematics and Information, Guangzhou University, Guangzhou, 510006 China; 2 Guangdong Provincial Engineering Technology Research Center for Wastewater Management and Treatment, School of Environment, South China Normal University, Guangzhou, 510006 China; 3 Guangzhou Sewage Purification Co. Ltd., Guangzhou, 510655 China; 4 Guangzhou Newtech Environment Technology Co. Ltd., Guangzhou, 510220 China

**Keywords:** Environmental sciences, Environmental chemistry

## Abstract

Co-combustion of municipal excess sludge (ES) and coal provides an alternative method for disposing ES. The present study aims to investigate the residual and ecological risk of heavy metals in fly ash from co-combustion of ES and coal. The total concentration and speciation distribution of heavy metals, characterization of SEM, EDX, XRD and leaching test were carried out to assess the fly ash in this study. The results showed that the total concentrations of Cu, Zn and Mn were higher than others in fly ash, and most heavy metals were concentrated in fine particles. For Cd, Cr and Pb, the percentages of speciation of F4 and F5 were all over 90%, suggesting the relatively lower leaching toxicity. The leaching percent of all heavy metals was lower than 5% by two diluted HNO_3_ solutions for fly ash. The potential ecological risks increased with the decrease of particle size of fly ash, and Cd accounted for the main fraction for ecological risk despite of lower concentration in comparison to other measured heavy metals.

## Introduction

The biological wastewater treatment leads to considerable amounts of excess sludge (ES) production, which must be treated and disposed of due to the increasingly stringent environmental regulations^1^. The production of municipal ES increases with the increasing wastewater treatment amounts due to the rapid urbanization and industrialization in China. However, sludge treatment and disposal are extremely expensive, which may contribute to over 50% of the total costs of a wastewater treatment plant (WWTP)^2^. The high amounts of ES produced in wastewater treatment plants poses a problem related with its final disposal. Therefore, it is very important to find out a secure, reasonable and high-efficiency sludge utilization way^3,4^.

The most common disposal processes for ES include landfilling, agricultural application, incineration and pyrolysis^5,6^. Most of processes are becoming increasingly difficult to operate, due to land limitations and more restrictive regulations. The ES incineration, a waste to energy technology, can reduce the utilization of fossil fuels and the environmental pollution^7^. Generally, sludge is a kind of fuel with high ash content, high moisture content, high volatile content and low calorific value^8^, which restrict the direct combustion of sludge.

One of the most attractive ways for the disposal of sewage sludge is its co-combustion with coal, however, the main issue is that heavy metals can be enriched in fly ash caused by co-combustion^9–11^. And the fly ash belongs to hazardous wastes and must be treated to avoid secondary pollution^12^. Wang et al.^13^ studied combustion behaviors and kinetics of sewage sludge blended with pulverized coal with and without catalysts and found that CeO_2_ and Fe_2_O_3_ showed good catalytic effects for the blended fuel in terms of comprehensive combustion performance and activation energy. Zhou et al.^14^ investigated the content and species distribution of heavy metals in MSWI fly ash with different particle size, and found that the volatile metals, including Zn, Pb, Cu and Cd, were easily concentrated in the fine particles exhibiting a great health risk. Barbosa et al.^15^ examined the co-combustion of coal and sewage sludge on chemical and ecotoxicological properties of ashes. Lin et al.^16^ examined the heavy metals residual in the incineration slag of textile dyeing sludge and found a negative correlation between the residual ratio of the heavy metals and the volatility during the incineration process.

Most researchers mainly focused on the combustion characteristics, heavy metals migration and species variation during co-combustion. However, there was few researches on the residual of heavy metals in fine particles and its potential ecological risk for subsequent final disposal. Moreover, it is necessary to exploit more reasonable technologies suitable for Chinese national conditions. In this study, the residual of heavy metals in fly ash with different particle size were examined through the determination of species of heavy metals, the characterization of SEM, EDX, XRD and anions content of fly ash were also investigated, and the potential ecological risk of residual heavy metals in fine particles was also assessed. The results would be helpful for the application and ecological risk assessment of excess sludge incineration in China.

## Materials and methods

### Sample preparation

In this study, excess sludge was collected each week from secondary sedimentation tank of a local municipal wastewater treatment plant (Guangzhou, China), the plant receives about 70% of domestic sewage and 30% of industrial wastewater at an average flow of approximately 250,000 m^3^/day. The produced excess sludge from the plant was disposed through co-combustion with coal in a waste incineration power plant. The predried sludge sample was prepared at 105 ℃ in an air dry oven for 24 h in triplicate until the mass no longer changed. The sludge–coal mixtures were prepared in a sludge–coal ratio of 1:3. The fly ash used in this study was sampled from the bottom of precipitator ash, respectively.

### Analytical methods

The proximate analysis and ultimate analysis of ES and PC were determined by TGA2000 according to GB/T212-2008 and Vario EL cube according to ASTM D5373-08 and GB/T214-2007, respectively. The calorific values were measured by HKRL-4000B according to GB/T213-2008. The concentrations of Cl^−^ and SO_4_^2−^ were measured using Ion chromatography (Dionex Aquion, USA), the concentrations of HCO_3_^−^ and CO_3_^2−^ were measured with double-indicator neutralization titration method. The morphology characteristics of the fly ash samples were measured using a scanning electron microscopy (SEM, ZEISS, Sigma 300) at the typical accelerating voltage of 5 kV. The content of each element in fly ash was determined with a Schottky Field Emission SEM-Energy Dispersive Spectrometer (EDX, Hitachi, SU-70). X-ray diffraction (XRD) was carried out with a PANalytical XPERT-3 Powder diffractometer with copper Kα radiation operating at 40 kV and 40 mA in the 3°–80° scan range of 2θ°.

### Speciation of heavy metals

In order to study the forms of Cd, Cr, Cu, Mn, Ni, Pb and Zn in bituminous coal and ES samples, the modified (five steps) Tessier extraction procedure was used^17^. Details of the procedure are given in Table 1. The extraction was carried out in glass centrifuge tubes of 50 mL capacity with an initial mass of 1 g oven dried (105 ℃) fine fraction (< 1 mm) of the samples. Supernatants were measured using atomic absorption spectroscopy. Analyses were performed in triplicate.

**Table 1:** Method of metal speciation in coal and ES samples.

Fraction	Form of metal	Parameters of fractionation	Time of extraction (h)
F1	Exchangeable	1.0 M CH_3_COONH_4_, pH 7.0, T = 20 °C	2.0
F2	Carbonate bound	1.0 M CH_3_COONa, pH 5.0, T = 20 °C	4.0
F3	Fe/Mn oxides bound	0.04 M NH_2_OH·HCl in 25% CH_3_COOH (v/v), T = 95 °C	4.0
F4	Organic	0.02 M HNO_3_ + 30% H_2_O_2_ + 3.2 M CH_3_COONH_2_ in 20% HNO_3_ (v/v), pH 2.0, T = 85 °C	5.5
F5	Residual	10 M HNO_3_ + 30% H_2_O_2_, T = 100 °C	1.5

### Ecological risk assessment method

The ecological risk index (RI) has been used to evaluate the degree of heavy metal contamination based on Eqs. (1) and (2)^18,19^.

1 $$ E_{i} = \frac{{{\text{T}}_{i} \times C_{i} }}{{C_{0} }} $$

2 $$ RI = \sum E_{i} $$

where *E*_*i*_ represents the monomial potential ecological risk factor for each heavy metal; *T*_*i*_ represents the toxic factor of each heavy metal; *C*_*i*_ and *C*_*0*_ represent the measured content and background reference value for each heavy metal, respectively. As shown in Table 2, RI stands for the potential ecological risk index for each of fly ash sample with different particle size. The *T*_*i*_ values used for calculation of RI for individual metal are Cu (5), Pb (5), Ni (5), Mn (1), Cr (2), Zn (1) and Cd (30)^18,20^. In order to accurately assess the potential ecological risk of each heavy metal, the bioavailable fractions (F1 + F2 + F3) of each heavy metal were used to calculate *E*_*i*_ in this study.

**Table 2:** Method of ecological risk assessment for fly ash.

*E*_*i*_	*RI*	Risk level
≤ 40	≤ 150	Low risk (LR)
41–80	151–300	Moderate risk (MR)
80–160	301–600	Considerable risk (CR)
160–320	601–1200	High risk (HR)
> 320	> 1200	Very high risk (VHR)

## Results and discussion

### Proximate analyses and calorific values of materials

The technological properties (volatile matter, ash and fixed carbon) of both dried sludge and bituminous coal diverge widely, which may suggest some major differences between the corresponding combustion properties^21^. The proximate analyses of dried sludge and coal are shown in Table 3.

**Table 3:** Ultimate and proximate analyses of predried ES and bituminous coal.

Sample	Proximate analysis (wt%, ad)			LHV (MJ/kg)	Ultimate analysis (wt%, ad)				
	VM	A	FC		C	H	N	O	S
Predried sludge	38.15	1.86	1.69	7.38	29.43	2.38	0.60	7.29	0.20
bituminous coal	21.91	0.61	43.10	13.54	38.46	2.32	4.65	2.16	0.52

*VM* volatile matter, *A* ash, *FC* fixed carbon.

The ash yield of bituminous coal is similar to that of dried ES sample. And the ES sample yields higher amount of volatiles (38.15 wt%, ad), while the bituminous coal sample yields only 21.91 wt%, ad. Apparently, dried ES sample shows much lower fixed carbon value than bituminous coal. The low heating value (LHV) of the dried ES sample on dry basis is 7.38 MJ/kg, while the LHV value of bituminous coal sample is 13.54 MJ/kg.

Table 4 shows the contents of heavy metals of predried excess sludge and bituminous coal. The results indicated that the contents of heavy metals in the predried sludge often outweigh those in bituminous coal, and the contents of heavy metals in the predried sludge often outweigh those in bituminous coal, and the Mn, Zn and Cu contents in the predried sludge were relatively higher than others, which was normally because of the influent of industrial wastewater. The contents of heavy metals in bituminous coal were all lower than those in sludge.

**Table 4:** Heavy metals contents of predried ES and bituminous coal (mg/kg).

Parameter	Cu	Pb	Ni	Mn	Cr	Zn	Cd
Predried sludge	1173.9	594.9	108.6	1762.1	624.0	1523.9	7.9
Bituminous coal	15.6	32.1	17.8	54.1	13.2	72.4	1.4

### Particle size distribution in fly ash

**Figure 1:**
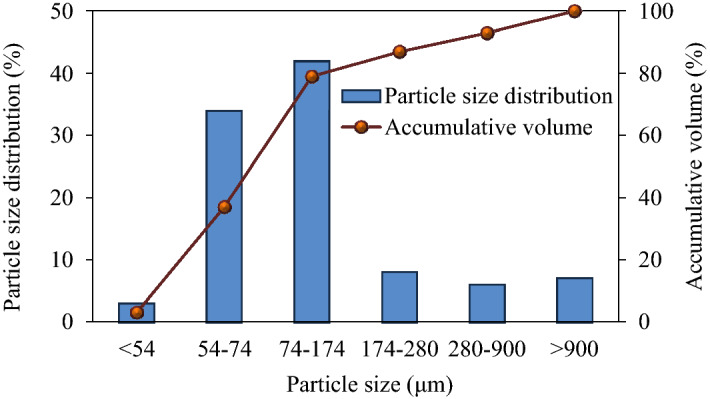
Particle size distribution of fly ash.

Figure 1 shows the distribution of particle sizes in fly ash. The results showed that the highest fraction of particle sizes within the range of 74–174 μm accounted for 42% of total particles in fly ash, the second fraction of particle sizes within the range of 54–74 μm accounted for 34% of total particles, and the fractions of other particle sizes were all lower than 10%.

### Total content and speciation of heavy metals in fly ash

**Figure 2:**
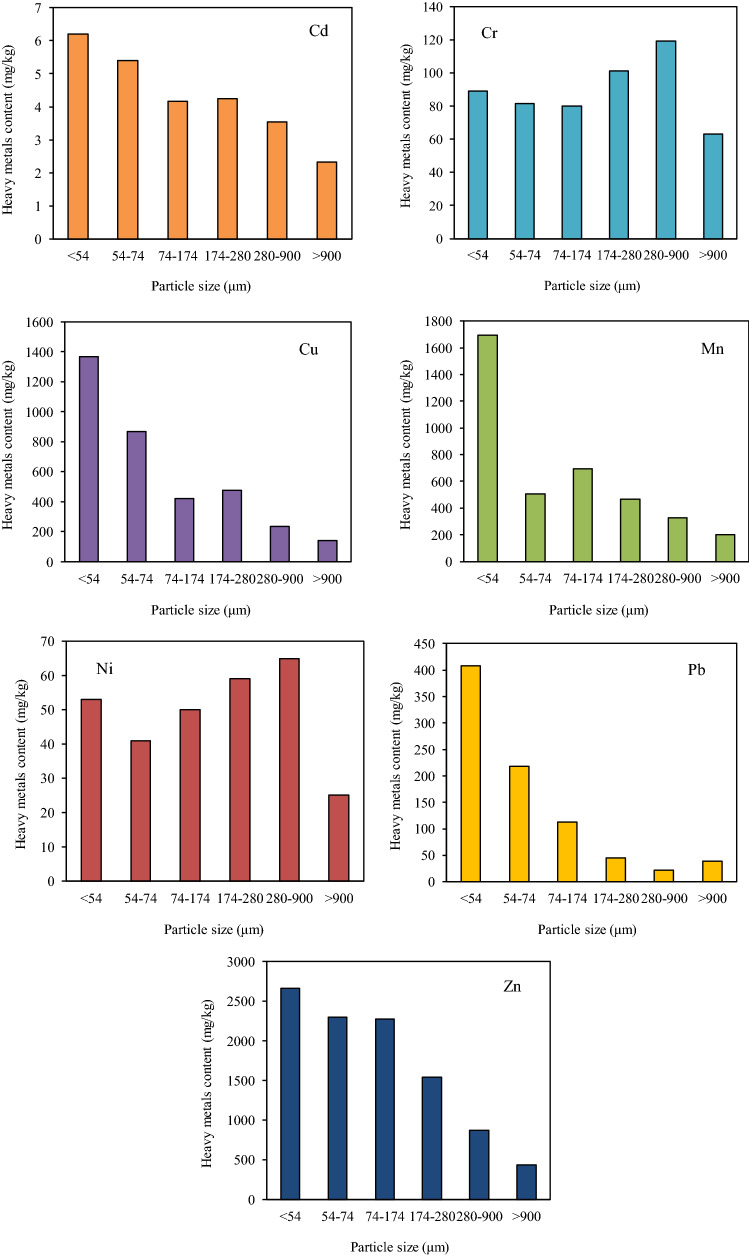
The total content and percentage of seven heavy metals in fly ash with different particle size (μm).

Figure 2 shows the distribution of seven heavy metals in fly ash with different particle sizes and their contribution to total content of heavy metals in fly ash. The higher contents of Cu, Zn and Mn were determined in fly ash, and mostly concentrated in fine particles with particle size lower than 54 μm. The results showed that the Cd content in fine particle was higher than those in coarse particles and middle particles, which can be attributed to low boiling point of Cd (765 ℃), resulting in the volatility during incineration. The formation mechanisms for fine particles and coarse particles were homomorphic condensation nucleation and heterophase condensation, respectively^22^. The distribution of Cu, Mn, Zn and Pb was also found similar to that of Cd, and the concentration was negatively correlated to particle size. Mn had a high boiling point (2097 ℃) and was stable during incineration. The highest Mn content was about 1700 mg/kg in the fine particles of lower than 54 μm. As for lithophilic heavy metals of Ni and Cr, there was little correlation between the contents of Ni and Cr and particle size, which can be attributed to higher boiling point of Ni (2837 ℃) and Cr (2672 ℃). However, due to the dramatic turbulence in the incinerator, part of heavy metals was enwrapped in the suspended particles in flue gas and then was captured in flue gas cleaning system, meanwhile, fine ash had little effect on condensation adsorption of Ni and Cr due to their low volatility, resulting in the uneven distribution of Ni and Cr in fly ash.

**Figure 3:**
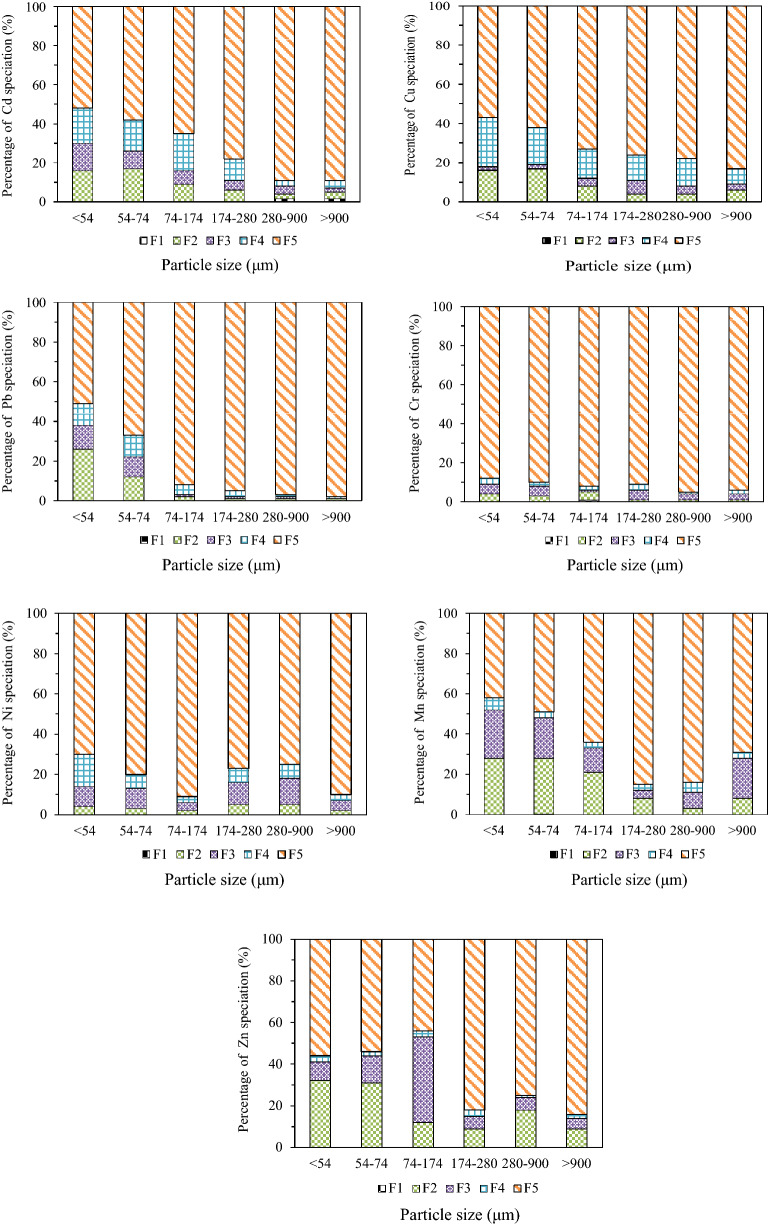
Speciation distribution and percentage of seven heavy metals in fly ash with different particle sizes (μm).

The ecotoxicity of heavy metals can be attributed to both concentrations and bioavailability^23^. Based on the modified Tessier extraction method, there are five speciations of heavy metals in fly ash. Heavy metals in exchangeable from (F1), carbonate bound form (F2) and Fe/Mn oxides bound form (F3) are usually considered to be mobile and bioavailable, however, heavy metals in organic form (F4) and residual form (F5) are generally stable and non-bioavailable^24^. As shown in Fig. 3, for Cd speciation distribution, the percentage of mobile and bioavailable forms mainly in F2 and F3 decreased with the increase of particle size in fly ash, and for the fly ash of particle size over 280 μm the percentage of relatively stable and non-bioavailable forms in F4 and F5 was over 92%, which suggested that Cd might show lower leaching toxicity.

As for Cu speciation distribution, there were only four speciation forms (F2, F3, F4 and F5) in fly ash. The percentage of mobile and bioavailable forms (F2 and F3) was about 18% for the particle size lower than 74 μm, and the percentage of mobile and bioavailable forms decreased to lower than 12% for the particle size over than 74 μm. The high stability of Cu-organic matter complexes was also found^24^. For Pb speciation distribution, the particle size lower than 54 μm showed the highest bioavailability of 38% in the forms of F2 and F3, and the percentage of F2 and F3 decreased to 22% for the particle size within 54–74 μm, however, the non-bioavailable forms in F4 and F5 increased to over 97% for the particle size over than 74 μm.

For Cr speciation distribution, there existed no obvious difference among all particle sizes of fly ash, the percentage of non-bioavailable form F5 was all over 88%. It was reported that the main Cr speciation in sludge were the organic and residual fractions^25^. For Ni speciation distribution, the percentage of stable and non-bioavailable forms of F4 and F5 accounted for over 82% for different particle sizes, which indicated that Cr and Ni had low leaching toxicity.

While for Mn speciation distribution, the percentage of mobile and bioavailable forms (F2 and F3) was in the range of 48–52% for the particle size lower than 74 μm, and the percentage of F2 and F3 decreased to lower than 12% for the particle size in the range of 174–900 μm. For Zn speciation distribution, the particle size lower than 174 μm showed relatively higher mobility, especially for the particle size in the range of 74–174 μm the percentage of speciation F2 and F3 reached up to 56%, however, those percentages of speciation F2 and F3 were all lower than 24% for particle size over than 174 μm.

### Characterization of SEM, EDX, XRD and anions content of fly ash

**Figure 4:**
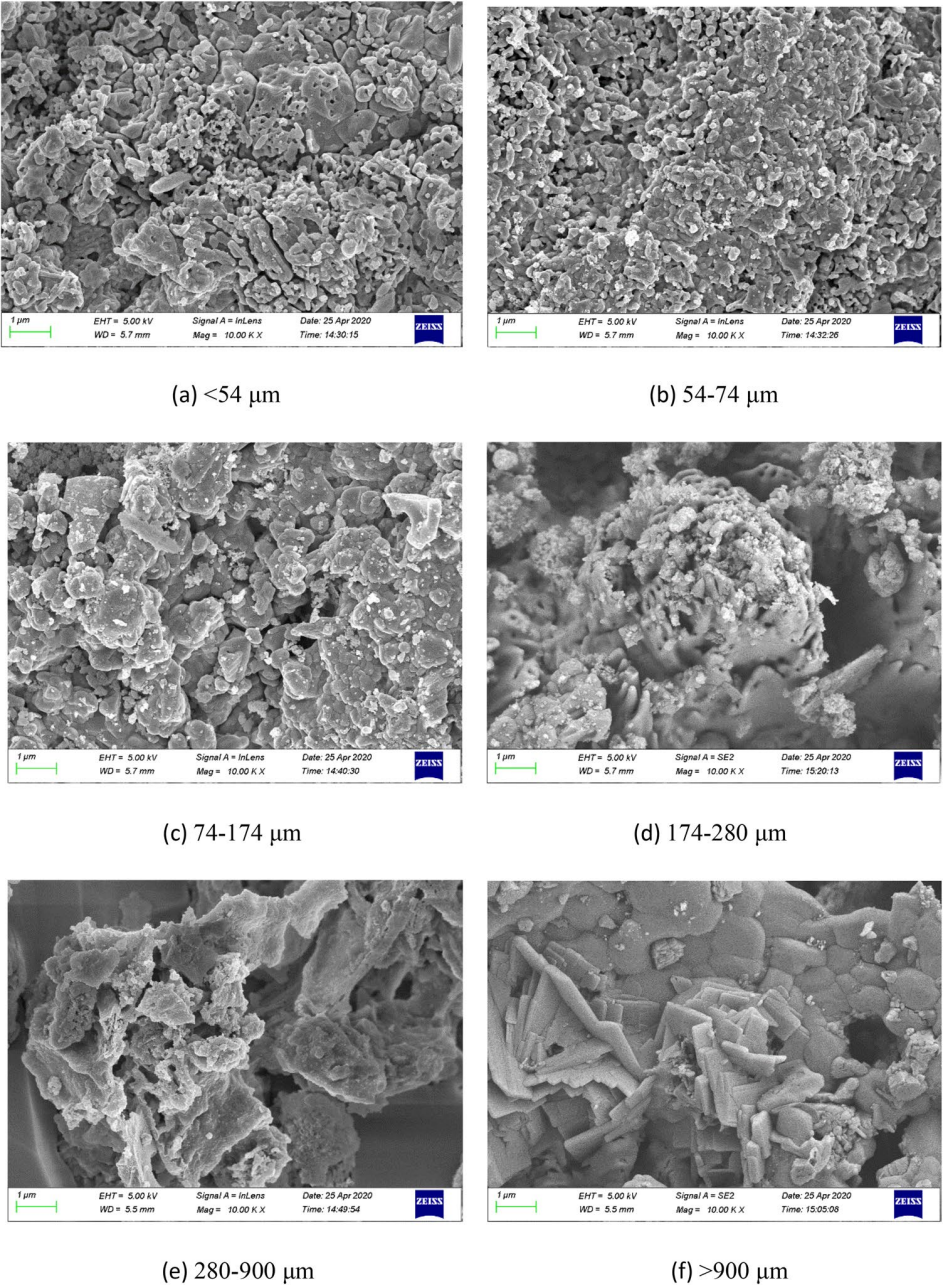
SEM micrographs of fly ash samples with different particle sizes.

Figure 4 shows the SEM micrographs of fly ash samples with different particle sizes. It can be observed that fine particles had more porosities for Fig. 4a (< 54 μm) and Fig. 4b (54–74 μm) of fly ash samples, which mainly consisted of homogeneously dispersed irregular particles in structure. While with the increase of particle size, there existed bulky grain aggregates on the surface of fly ash samples, especially for the particles over than 280 μm, nearly no dispersed fine particles were found on the outer surface of fly ash samples.

**Figure 5:**
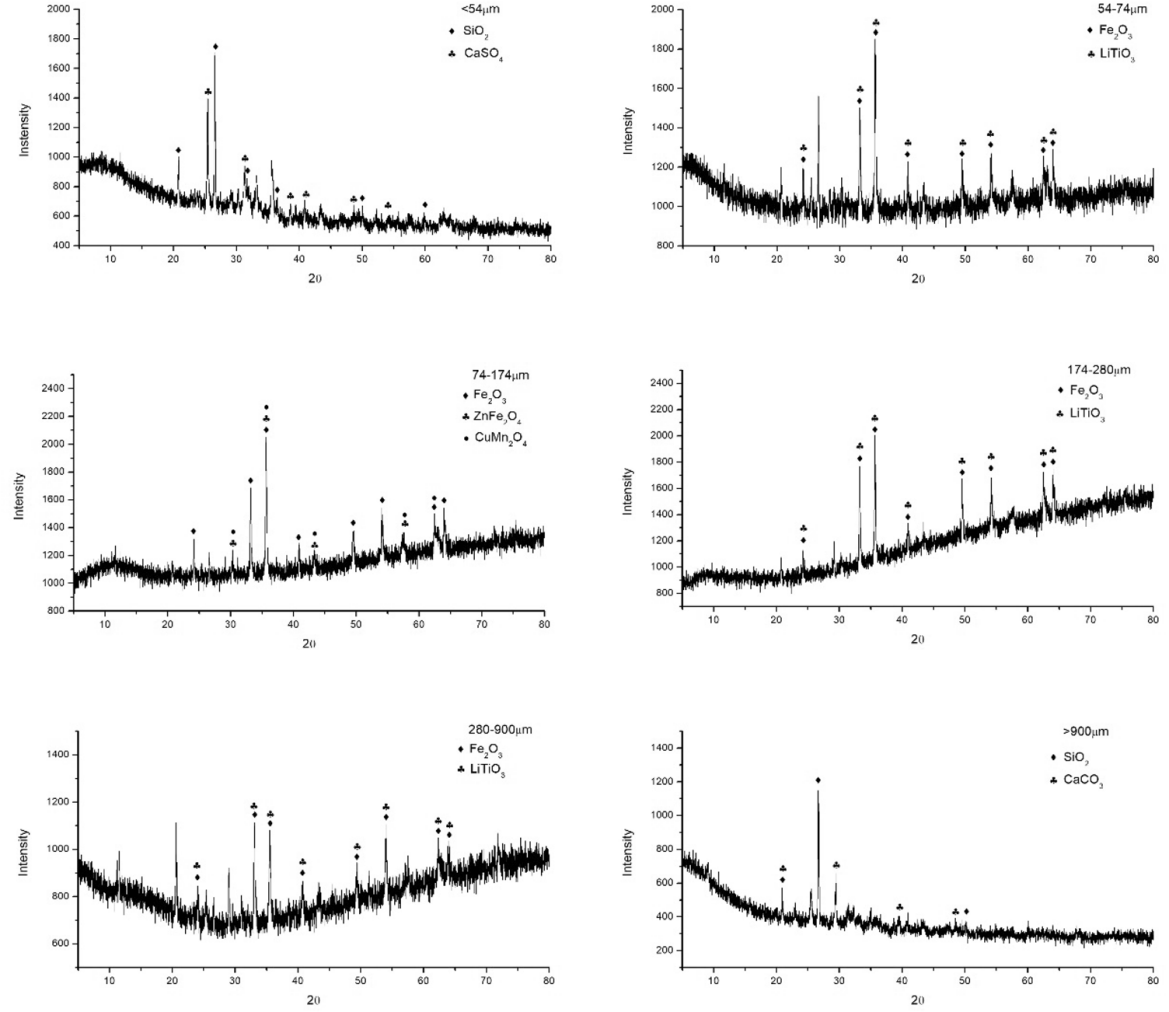
XRD spectrums of fly ash samples with different particle sizes.

Table 5 shows the EDX results of fly ash samples with different particle sizes. And the XRD spectrums of fly ash samples are shown in Fig. 5. High contents of oxygen (O) and ferrous (Fe) were found in fly ash based on EDX analysis, which indicated the possible presence of ferric oxide. On the other hand, according to the XRD results, the presence of ferric oxide was confirmed for the particles of within 54–900 μm (Fig. 5). In addition, there also existed carbon (C), silicon (Si), calcium (Ca), alumium (Al) and sulfur (S) in fly ash, and three crystalline compounds, including silicon dioxide (SiO_2_), calcium sulfate (CaSO_4_) and calcium carbonate (CaCO_3_), were also found in the XRD spectrums of fly ash (Fig. 5).

**Table 5:** EDX analysis of fly ash samples with different particle sizes.

	< 54 μm	54–74 μm	74–174 μm	174–280 μm	280–900 μm	> 900 μm
C (at.%)	11.75	9.60	14.77	25.50	8.45	12.85
O (at.%)	58.30	46.33	48.38	45.91	54.73	54.26
Mg (at.%)	2.81	BDL	BDL	BDL	3.93	0.60
Al (at.%)	1.67	1.17	0.85	1.99	1.95	7.11
Si (at.%)	20.67	0.79	0.23	0.36	1.40	3.61
P (at.%)	0.33	0.20	BDL	0.17	0.26	2.22
S (at.%)	0.69	0.11	BDL	0.14	0.23	3.33
K (at.%)	0.18	BDL	BDL	BDL	0.09	1.08
Ca (at.%)	1.87	0.23	0.11	0.30	0.40	1.71
Fe (at.%)	1.60	20.10	27.80	22.54	21.96	3.47
F (at.%)	BDL	BDL	BDL	BDL	BDL	8.83
Cl (at.%)	BDL	BDL	BDL	BDL	BDL	0.13

*BDL* below detection limit.

**Figure 6:**
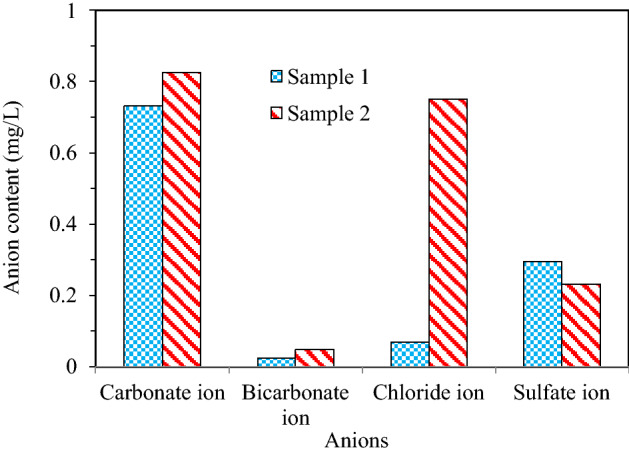
Anions content in fly ash.

Little chlorine content was measured by the EDX results, which was close to the results from the determination of anions content (Fig. 6). Based on the XRD spectrums, the crystalline compounds of LiTiO_3_, ZnFe_2_O_4_ and CuMn_2_O_4_ were also determined, however, the contents of Zinc (Zn), copper (Cu), manganese (Mn) were below the detection limit (0.1%) in the EDX results.

The contents of water-soluble salts in the collected fly ash samples are shown in Fig. 6. The anions contents, including Cl^−^, SO_4_^2−^, CO_3_^2−^ and HCO_3_^−^, were determined to examine the effect of anions on the residue of heavy metals in fly ash. The heat reduction rate of the collected fly ash samples was in the range of 1.16–1.58%, which indicated that the content of combustible substances (including organic matter) in fly ash was low, meeting the requirements of related resource utilization. For the fly ash samples, the total salt contents of the two fly ash samples were 1.41 and 2.02 mg/L, respectively. And the main difference between two samples existed for the chlorine ion content. The low anion contents in fly ash would not cause soil salinization when considering subsequent disposal of the incineration residues^26^.

### Leaching test of heavy metals

**Figure 7:**
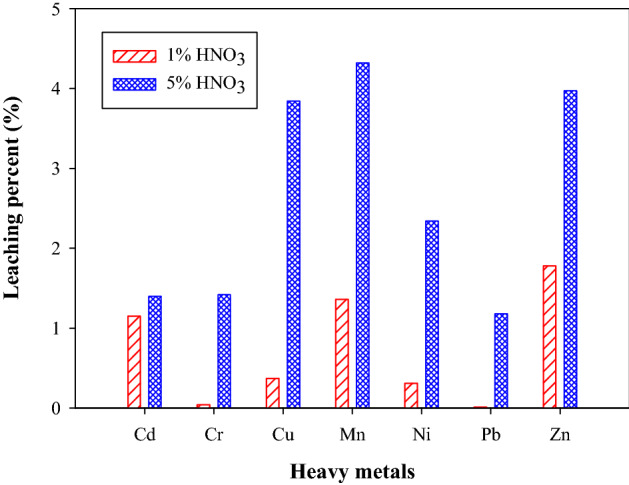
Leaching percent of heavy metals from fly ash.

The leaching properties of heavy metals from fly ash by diluted HNO_3_ are shown in Fig. 7. The leaching percent of all heavy metals was lower than 5% for two diluted HNO_3_ solutions. According to sequential extraction test, the contents of carbonate-bound fraction (F2) and iron oxides bound fraction (F3) were higher for heavy metals of Cu, Mn, Ni, Zn, therefore, the corresponding leaching percent was relatively higher than three other heavy metals. The HCl extraction would dissolve more soluble oxides and carbonate^27^, which was similar to the leaching test by diluted HNO_3_ in this study. And the contents of residual fraction were higher for heavy metals of Cd, Cr and Pb, which resulted in lower leaching percent by diluted HNO_3_.

### Ecological risk assessment of heavy metals in fly ash

Table 6 shows the *E*_*i*_ and RI values of seven heavy metals of Cd, Cr, Cu, Mn, Ni, Pb and Zn. It was shown that the *E*_*i*_ values of heavy metals decrease with the increase of particle size. The *E*_*i*_ values for Cr, Cu, Mn, Ni, Pb and Zn were all less than 40, indicating low risk based on the classification of risk level (Table 2). While Cd in fly ash with lower than 74 μm was classified as moderate risk, the *E*_*i*_ values of Cd in fly ash with other particle sizes were all lower than 40, indicating low risk.

**Table 6:** Comparison of E_i_ and RI values for heavy metals in fly ash samples.

Samples	*E*_*i*_							*RI*
	Cd	Cr	Cu	Mn	Ni	Pb	Zn	
< 54 μm	55.8	0.2	24.6	11.0	0.9	11.1	6.2	109.8
54–74 μm	42.0	0.1	16.5	3.0	0.7	3.4	5.8	71.6
74–174 μm	20.0	0.1	5.1	2.9	0.4	0.2	6.9	35.5
174–280 μm	14.0	0.1	5.3	0.7	1.2	0.1	1.3	22.6
280–900 μm	8.5	0.1	1.9	0.4	1.5	0.0	1.2	13.6
> 900 μm	4.9	0.1	1.3	0.7	0.2	0.0	0.3	7.5

The overall potential ecological risks of seven heavy metals are shown in Table 6. The highest RI value of fly ash sample with particle size lower than 54 μm was 109.8, indicating low risk, which may be mainly due to Cd and Cu^15^. The other fly ash samples with different particle size were all classified as low risk. The bioavailable fractions of each heavy metal were used to calculate *E*_*i*_ in this study, according to Table 6, Cd with the highest *T*_*i*_ value (30) among tested metals, was enriched in fine particles, and accounted for the main fraction for ecological risk despite of lower concentration in comparison to other heavy metals measured. Therefore, the potential ecological risks increased with the decrease of particle size of fly ash.

## Conclusion

The total concentrations of Cu, Zn and Mn were higher than other metals in fly ash, and most heavy metals were concentrated in fine particles. The potential ecological risks increased with the decrease of particle size of fly ash, and Cd accounted for the main fraction for ecological risk despite of lower concentration in comparison to other measured heavy metals. Therefore, considering land application of fly ash from co-combustion of ES and coal, Cd would be a main concern.
